# Visualizing weather radar data from volcanic eruption clouds

**DOI:** 10.1016/j.dib.2021.106942

**Published:** 2021-03-06

**Authors:** Masayuki Maki, Yura Kim

**Affiliations:** a Research and Education Center for Natural Hazards, Kagoshima University, Kagoshima 890-0065, Japan; b Weather Radar Center, Seoul 156-720, Republic of Korea

**Keywords:** ANT3D, Computer graphics. DIAS, Matlab, Sakurajima, Visualization

## Abstract

This paper is submitted to accompany the article “Analyses of three-dimensional weather radar data from volcanic eruption clouds” [1]; it describes three-dimensional (3D) visualizations of the Sakurajima volcanic eruption clouds and the weather radar data used for analyses, as well as their availability and downloading procedures. The radar data were acquired by an operational X-band weather radar located approximately 11 km south of the Showa vent of Sakurajima in Kagoshima, Japan. The original raw radar data are available from the “XRAIN Precipitation Original Data search and Download System”, which is hosted on the website “Data Integration and Analysis System (DIAS)”. Animated images of the radar data shown here, which provide a visual explanation of the temporal evolution and the inner structure of volcanic eruption clouds, were created using the program “Analysis Tools of Three-dimensional Weather Radar Data (AN3D)”. The detailed methods of ANT3D are provided in the co-submitted article “Construction of three-dimensional weather radar data from volcanic eruption clouds” [2].

## Specifications Table

**Table:** 

Subject	Earth and Planetary Sciences
Specific subject areas	Monitoring of volcanic eruptions/Volcanology Data visualization/Radar meteorology
Types of data	Table Figure Image
How data were acquired	Volcanic eruption cloud radar data were acquired by an X-band polarimetric weather radar operated by the Ministry of Land, Infrastructure, Transport and Tourism (MLIT). The radar data were visualized in three dimensions using the Analysis Tools of Three-dimensional Weather Radar Data (ANT3D) system developed by Kagoshima University.
Data formats	Raw radar data: original data format Visualized data: animation gif, MP4
Parameters for data collection	Temporal resolution: 5 min Spatial resolution: 150 m and 1.0° in the range and azimuthal directions, respectively
Description of data collection	Radar data were collected by MLIT; they are archived in the Data Integration and Analysis System (DIAS) and can be accessed via the XRAIN Precipitation Original Data search and Download System.
Data source location	Volcanic eruption cloud data: Sakurajima Showa vent, Kagoshima, Japan
Data accessibility	Raw radar data are available from: https://auth.diasjp.net/cas/login?service=http://xrain.diasjp.net/original/&locale=en A user ID and password can be requested by contacting dias-office@diasjp.net Visualized data are available from Mendeley Data (http://dx.doi.org/10.17632/spr2pbzzzw.2).
Related research article	Maki, M., Kim, Y., Kobori, T., Hirano, K., Lee, D.-I., Iguchi, M., 2021. Analyses of three-dimensional weather radar data from volcanic eruption clouds. J. Volcanol. Geotherm. Res. (co-submitted; https://doi.org/10.1016/j.jvolgeores.2021.107178). [1]

## Value of the Data

• These data demonstrate the usefulness of weather radar monitoring of eruption clouds.
• These data will be of interest to volcanologists, volcanic disaster prevention officials, and students of volcanic eruption cloud dynamics.
• Polarimetric radar parameters for eruption clouds are useful for studying microphysical processes in eruption clouds.
• Sakurajima eruption cloud radar data accumulated during the past decade have facilitated statistical analyses of eruption cloud dynamics.

## 1 Data Description

### 1.1 List of explosive volcanic eruptions

**Fig. 1:**
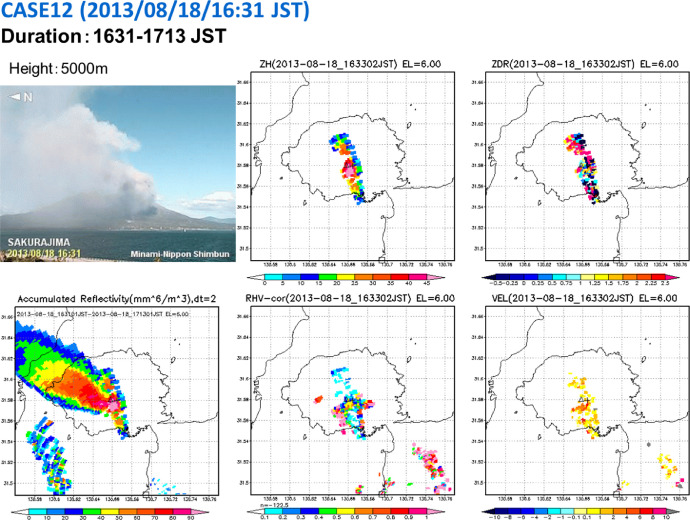
An example of Plan Position Indicator (PPI) images of temporally integrated reflectivity factors for the 31 Sakurajima volcanic eruption clouds.

According to the Japan Meteorological Agency, Sakurajima erupted 1097 times in 2013. Among these eruptions, we selected 31 cases from DIAS data to analyze the structure of volcanic eruption clouds. All selected eruption cases had an eruption cloud top height ≥ 3000 m above the vent. Information on these 31 eruptions are provided in Table 1, including eruption type and eruption onset time; ash cloud top height, direction, and duration; and ash fall amount. Images of temporally accumulated reflectivity factors for the 31 volcanic eruption clouds are available for download from Mendeley Data [3]. An example of images is shown in Fig. 1.

**Table 1:** Details of 31 Sakurajima volcanic eruptions in 2013 selected for analysis. In all cases, the eruption cloud height was ≥3000 m above the vent. ERP, eruption; EXP, experiment; DIR, direction.

No	ERP ID	EXP ID	ERP Time (JST)	ERP Time (UTC)	Duration (UTC)	Am	Height (m)	DIR	Vent
1	369	305	2013/05/08 16:27:07	2013/05/08 07:27:07	07:25–08:21	4	3300	T	S
2	421	352	2013/06/13 08:58:44	2013/06/12 23:58:44	23:57–01:59	4	3000	T	S
3	423	354	2013/06/13 13:26:04	2013/06/13 04:26:04	04:25–05:13	4	3300	NE	S
4	458	380	2013/07/10 17:58:09	2013/07/10 08:58:09	08:58 −09:23	4	3400	T	S
5	461	382	2013/07/11 13:06:24	2013/07/11 04:06:24	04:05–04:53	4	3000	N	S
6	488	–	2013/07/16 15:56:–	2013/07/16 06:56:–	06:55–07:25	4	3500	NE	S
7	509	411	2013/07/19 07:37:06	2013/07/18 22:37:06	22:35–23:19	4	3400	SE	S
8	511	413	2013/07/19 12:04:40	2013/07/19 03:04:40	03:03–04:01	4	3000	T	S
9	531	428	2013/07/22 16:35:–	2013/07/22 07:35:–	07:35–08:31	4	3200	E	S
10	535	431	2013/07/22 23:33:52	2013/07/22 14:33:52	14:33–15:13	4	3000	E	S
11	596	478	2013/08/09 12:50:42	2013/08/09 03:50:42	03:49–04:49	4	3500	SE	S
12	627	500	2013/08/18 16:31:05	2013/08/18 07:31:05	07:31–08:13	5	5000	NW	S
13	637	509	2013/08/21 10:06:54	2013/08/21 01:06:54	01:05–01:33	4	3500	NW	S
14	695	554	2013/08/29 09:27:–	2013/08/29 00:27:–	00:25–00:59	5	3000	E	S
15	731	–	2013/09/06 16:23:–	2013/09/06 07:23:–	07:23–07:51	4	3300	T	S
16	734	586	2013/09/06 20:44:10	2013/09/06 11:44:10	11:43–12:11	4	3000	N	S
17	762	603	2013/09/12 13:26:–	2013/09/12 04:26:–	04:27–04:59	4	3300	T	S
18	764	604	2013/09/13 07:29:51	2013/09/12 22:29:51	22:29–22:59	4	3000	T	S
19	811	–	2013/09/20 16:46:–	2013/09/20 07:46:–	07:45–08:19	4	3500	W	S
20	838	660	2013/09/25 12:42:59	2013/09/25 03:42:59	03:41–04:13	5	4000	S	S
21	842	–	2013/09/26 10:18:41	2013/09/26 01:18:41	01:17–01:55	5	4500	S	S
22	846	663	2013/09/27 17:24:07	2013/09/27 08:24:07	08:23–09:09	4	3000	T	S
23	867	680	2013/10/01 13:38:50	2013/10/01 04:38:50	04:39–05:17	4	3000	S	S
24	882	693	2013/10/03 08:30:08	2013/10/02 23:30:08	23:29–24:05	4	3000	E	S
25	886	696	2013/10/04 04:33:42	2013/10/03 19:33:42	19:17–20:45	4	3000	W	S
26	887	697	2013/10/04 12:02:53	2013/10/04 03:02:53	03:01–03:37	4	3000	W	S
27	895	702	2013/10/07 09:22:51	2013/10/07 00:22:51	00:21–00:41	4	3000	W	S
28	954	–	2013/10/21 10:35:–	2013/10/21 01:35:–	01:35–02:25	4	4500	T	S
29	972	757	2013/10/28 08:09:36	2013/10/27 23:09:36	23:09–23:51	4	3500	N	S
30	974	–	2013/10/28 12:39:–	2013/10/28 03:39:–	03:37–04:13	4	3200	N	S
31	1047	807	2013/11/24 16:23:01	2013/11/24 07:23:01	07:23–07:51	5	4000	NE	S

### 1.2 Raw radar data selected for three-dimensional analyses

Eruption cases listed in Table 1 can be used to study the inner structure of volcanic eruption clouds. In our co-submitted paper [1], we selected the following three cases from among those shown in Table 1 to study the effects of environmental wind on volcanic eruption cloud structure:

• Case 1 (week wind): Sakurajima eruption at 10:21 LST, June 13, 2013
• Case 2 (strong wind): Sakurajima eruption at 08:30 LST, October 03, 2013
• Case 3 (moderate wind): Sakurajima eruption at 16:31 LST, August 18, 2013

Raw radar data for these eruptions are available from the XRAIN Precipitation Original Data search and Download System [4,5] on the Data Integration and Analysis System (DIAS) website [6]. A user ID and password are required to access the download system.

### 1.3 Animation of radar images of volcanic eruption clouds

The raw radar data downloaded from DIAS were analyzed using the Analysis Tools of Three-dimensional Radar Data (ANT3D) program [2,7]. The Constant Altitude Plan Position Indicator (CAPPI) viewer was used for 3D visualization of the analysis results. These software programs can be downloaded from the Mendeley Data website [7]. Commercial visualization software packages [8,9] were also used for data visualization.

Three-dimensional structure of volcanic eruption clouds using programs mentioned above is shown in the article “Analyses of three-dimensional weather radar data from volcanic eruption clouds” [1]. The analyzed results are effectively visualized by five animations provided in the present paper. Animation 1 shows the comparison of observed Plan Position Indicator (PPI) images collected at 5 min intervals and temporally interpolated PPI images produced at 30 s intervals for an elevation angle of 7.5° Animation 2 shows comparison of observed PPI images for 12 tilt angles (1.7–20°) and elevation angle-interpolated PPI images produced at 0.5° intervals. These spatially and temporary interpolated PPI data are used to construct three-dimensional volcanic eruption clouds. Animation 3 shows the volume rendering of three-dimensional (3D) views of the volcanic eruption cloud observed east of the Sakurajima Showa vent at 16:41 LST, August 18, 2013. Animation 4 shows horizontal and vertical cross-sections of the same volcanic eruption cloud identical with that shown in Animation 3. Animation 5 shows temporal evolution of eruption clouds under calm wind (June 13, 2013), strong wind (October 07, 2013), and moderate wind (August 18, 2013). Animation files mentioned above can be found in the online version at doi:10.1016/j.dib.2021.106942.

Results mentioned above are also available for download from Mendeley Data [3].

**File: “2013_31cases_pdf” (935** **KB)**

This folder contains the list of details of 31 Sakurajima volcanic eruptions in 2013 selected for analysis (Table 1) and images of temporally accumulated reflectivity factors for the 31 volcanic eruption clouds (Fig. 1). In all cases, the eruption cloud height was ≥3000 m above the vent.

ERP, eruption; EXP, experiment; DIR, direction.

**File: “2013_31cases_mp4.zip” (288** **MB)**

The folder “2013_31cases_mp4” contains 31 animation files. Each file name is constructed as caseXX.mp4, where XX is the case number from 01 to 31. In each file, animated Plan Position Indicator (PPI) images of polarimetric radar parameters such as reflectivity (ZH), differential reflectivity (ZDR), correlation coefficient (RHV), and Doppler velocity (VEL) for an elevation angle of 6° are shown. The accumulated reflectivity [mm^6^ m^–3^] of each eruption is also shown. Web camera images of volcanic ash clouds were provided by Minami Nihon Shinbun and are also available from the database. X-band polarimetric radar data were provided by the Ministry of Land, Infrastructure, Transport and Tourism (MLIT).

**File: “3D_animation_mp4.zip”**

The folder “3D_animation_mp4” contains five animation files shown in the present paper, which helps to understand the results shown in the article “Analyses of three-dimensional weather radar data from volcanic eruption clouds” [1].

## 2 Experimental Design, Materials and Methods

### 2.1 Experimental area

**Fig. 2:**
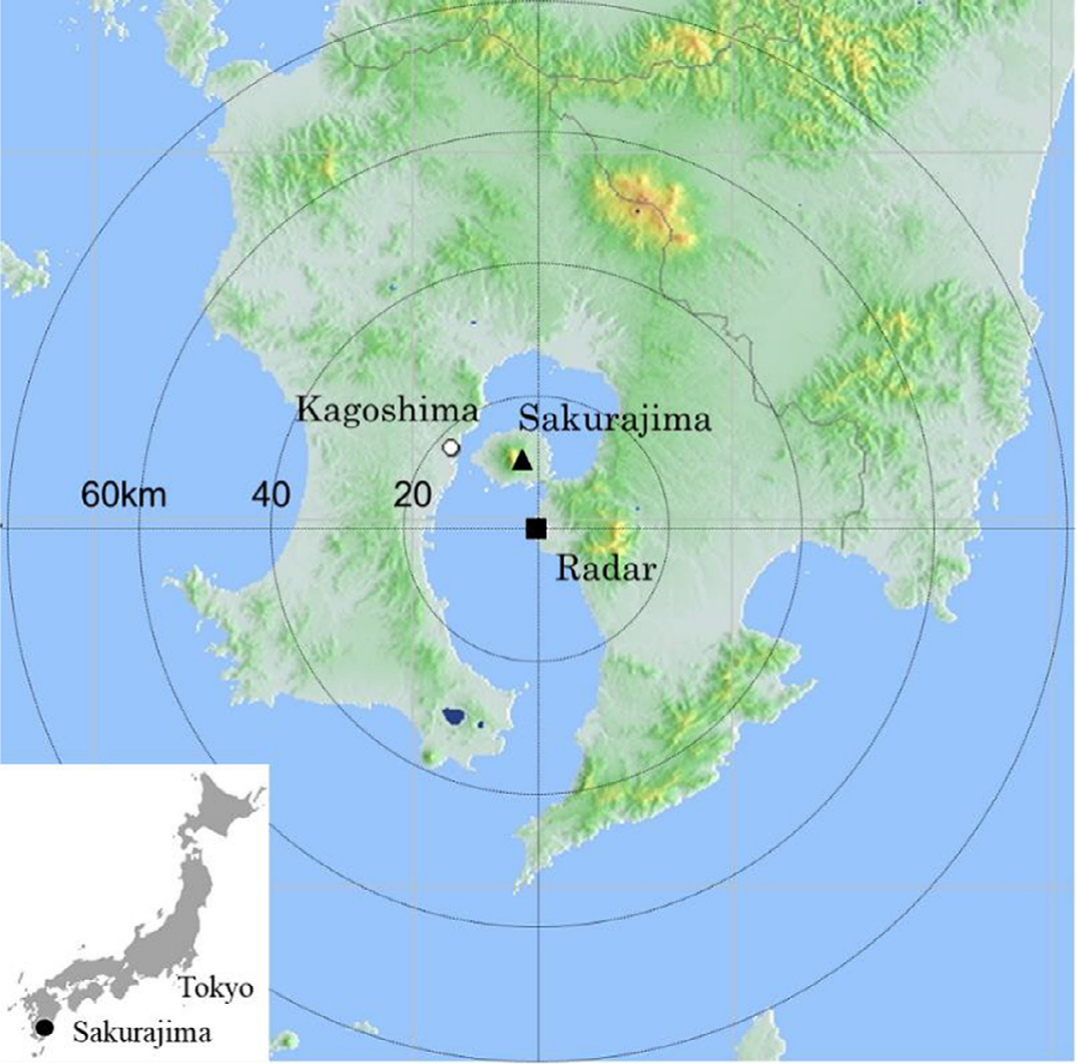
Map of Sakurajima volcano eruptions and X-band polarimetric weather radar.

The X-band polarimetric weather radar is located approximately 11 km south of the Sakurajima Showa vent (Fig. 2). This radar was established by MLIT in 2013 as a countermeasure against volcanic mudflows from Sakurajima. The main specifications and antenna scanning mode of the radar system are listed in Table 2.

**Table 2:** Main specifications of the X-band polarimetric radar.

Code name		TRM (Tarumizu)

Antenna	Diameter Beam width Gain Speed Scan angle, PPI	2.2 m 1.0° 44.7 dB (H), 45.1 dB (V) 1–4 rpm 1.7°- 20.0° (12 tilts)


Transmitter	Frequency Power PRF Pulse width	9770 MHz 200 W 1500/2000 pps 1.0 µs

Receiver	Smin	−109.5 dBm

Measured radar parameters		Z_h_, Z_v_, Z_DR_, Φ_DP,_ ρ_hv_, V_d_, σ
Resolution		Range: 150 m Azimuth: 1.0°

Z_h_:Reflectivity factor (horizontal polarization), Z_v_: Reflectivity factor (vertically polarization).

Z_DR_:differential reflectivity, Φ_DP,_:differential phase, ρ_hv_,:correlation.

V_d_:Doppler velocity, σ:spectrum width.

### 2.2 Available data

Two types of data are available from web sites: the image data of eruption clouds and the raw radar data. The former data includes three-dimensional animation images of eruption clouds obtained from the AT3D software. The latter data includes raw radar data such as reflectivity factor, Doppler velocity, and polarimetric radar parameters of eruption clouds. The image data is preferable for readers such as volcanologists, who are interested in the inner structure of eruption clouds and its temporal changes. The raw radar data are valuable for people who are specialized in radar meteorology and interested in developing algorithms of the quantitative ash fall estimation and radar data visualizations. It should be noted that analyzing raw radar data requires at least basic knowledge on meteorological radar.

### 2.3 Analysis of raw radar data

The GUI software `ant3d_gui' [7] developed by Kagoshima University is provided to researchers who are interested in analyzing raw radar data. The detailed explanations on the data quality controls and algorithms used in the ant3d_gui can be found in co-submitted articles [2,7]. In this subsection, we describe an outline of procedures necessary to analyze raw radar data. The following preparations are necessary before data analyses.

1. Preparation of the raw radar data: a user ID and password, that are necessary to access the data base and download the raw radar data, are requested by contacting dias-office@diasjp.net.
2. Preparation of software: programs ʹant3d_guiʹ and ʹcappiviewerʹ are available from Mendeley Data [7]. To run these program, download the MATLAB Runtime installer (release R2019a for Windows) from the MathWorks website [10] and install it on your computer.
3. Data analysis: fallow the user's manual of ʹant3d_guiʹ [2].

## Ethics Statement

Not applicable.

## CRediT Author Statement

**Masayuki Maki:** Conceptualization, Methodology, Writing - Original draft preparation, Reviewing and Editing; **Yura Kim:** Visualization, Software, Data curation, Investigation.

The English in this document has been checked by at least two professional editors, both native speakers of English. For a certificate, please see: http://www.textcheck.com/certificate/vhDVHq

## Declaration of Competing Interest

The authors declare that they have no known competing financial interests or personal relationships that could appear to have influenced the work reported in this paper.

## Appendix Supplementary materials

Animation 1. Comparison of (a) observed Plan Position Indicator (PPI) images collected at 5 min intervals and (b) temporally interpolated PPI images produced at 30 s intervals for an elevation angle of 7.5° Image, video 1

Animation 2. Comparison of (a) observed PPI images for 12 tilt angles (1.7–20°) and (b) elevation angle-interpolated PPI images produced at 0.5° intervals. Image, video 2

Animation 3. Volume rendering of three-dimensional (3D) views of the volcanic eruption cloud observed east of the Sakurajima Showa vent at 16:41 LST, August 18, 2013. Image, video 3

Animation 4. As in Animation 3, but for horizontal and vertical cross-sections of the 3D volcanic eruption cloud. Image, video 4

Animation 5(a). Temporal evolution of eruption clouds under weak wind conditions (June 13, 2013). Image, video 5

Animation 5(b). Temporal evolution of eruption clouds under strong wind conditions (October 07, 2013). Image, video 6

Animation 5(c). Temporal evolution of eruption clouds under moderate wind conditions (August 18, 2013). Image, video 7
